# Decreased circulating omega-3 fatty acids increase the risk of myocardial infarction: a two-sample Mendelian randomization study

**DOI:** 10.3389/fcvm.2024.1328087

**Published:** 2024-03-14

**Authors:** Wei Wang, Linfei Yang, Jing Zhang, Haiyun Xiang

**Affiliations:** ^1^ Department of Cardiology, The Second People’s Hospital of Hefei, Hefei, Anhui, China; ^2^ Department of Cardiology, Hefei Hospital Affiliated to Anhui Medical University, Hefei, Anhui, China; ^3^ Infant Care Services and Management, Institution of Culture Tourism and Education, Anhui Technical College of Industry and Economy, Hefei, Anhui, China

**Keywords:** omega-3 fatty acids, myocardial infarction, Mendelian randomization study, SNPs, instrumental variables

## Abstract

**Background:**

Many studies have shown that omega-3 fatty acids may play critical roles in cardiovascular diseases. Myocardial infarction (MI) typically results from a thrombotic occlusion of a coronary artery leading to myocardial ischemia. Thus, this study aims to examine the association between omega-3 fatty acids and MI.

**Methods:**

A two-sample Mendelian randomization study was used to explore the causal relationship between circulating omega-3 fatty acids and the risk of MI performed by MR-Egger regression, inverse-variance weighted (IVW), weighted median, and weighted mode.

**Results:**

Five single-nucleotide polymorphisms strongly related to circulating omega-3 fatty acids were selected as instrumental variables from a published genome-wide association study (GWAS) meta-analysis including 13,544 subjects. We extracted summary data for the risk of MI from another GWAS meta-analysis including 171,875 individuals (43,676 cases and 128,199 controls). The genetically predicted lower circulating omega-3 increased the risk of myocardial infarction showed by the results of IVW [odds ratio (OR) = 1.224, 95% CI = 1.045–1.433, *P* = 0.012], weighted median method (OR = 1.171, 95% CI = 1.042–1.315, *P* = 0.008), and weighted mode (OR = 1.149, 95% CI = 1.002–1.317, *P* = 0.117), although the result of MR-Egger was not significant (OR = 0.950, 95% CI = 0.513–1.760, *P* = 0.880) with a wider confidence interval.

**Conclusion:**

The findings from our Mendelian randomization analysis suggest that the association between omega-3 fatty acid levels and MI is likely causal.

## 1 Introduction

Omega-3 (n-3) fatty acids are a family of polyunsaturated fatty acids (PUFA) that cannot be produced in mammals. The main members of omega-3 fatty acids are eicosapentaenoic acid (EPA) and docosahexaenoic acid (DHA), which must be obtained from the ocean, and linolenic acid, which is obtained from vegetable oils. Omega-3 fatty acids are good for the secondary prevention of coronary artery disease and stroke. The most functionally important n-3 PUFA, EPA and DHA, exist in fish and other seafood (1).

Many long-term prospective cohort studies and meta-analyses have provided supportive evidence that higher intakes of EPA and DHA reduce the risk of developing cardiovascular disease (CVD) in the general population (2, 3). Zhang et al. found the significant association between higher n-3 PUFA intake and decreased total mortality (4). A total of 54,230 men and 30,882 women died during the 6.07 million person-years of follow-up. Compared to the lowest quintiles of fish intake, males and females with the highest quintiles of fish intake had 10% (6%–15%) and 10% (3%–17%) lower CVD mortality, respectively (4).

Acute myocardial infarction (AMI) is a common cardiovascular event that results from a reduction or interruption of blood flow to a portion of the heart, leading to necrosis of heart muscle. Myocardial infarction (MI) leads to a large number of deaths and disabilities and carries a significant socio-economic burden (5). AMI can result in the development of heart failure although the ischemic episode is often not fatal (6). Many studies have studied the relationships between MI and omega-3 fatty acids. Omega-3 fatty acids are believed to protect against MI through potential multiple mechanisms. They reduce triglyceride levels (7), diminishing the risk of atherosclerosis, and exhibit anti-inflammatory properties, lowering the inflammatory response associated with cardiovascular diseases (8). In addition, these acids enhance heart rate variability and decrease platelet aggregation, reducing thrombosis and further mitigating the risk of MI (3). The synergy of these mechanisms underscores the significant role of omega-3 in cardiovascular prevention. However, many trial studies were short in duration and concentrated on patients who had preexisting coronary heart disease (CHD) or high risk for CHD (9), and the background dietary intake of subjects in these trials was unknown, which could bias the results. Therefore, the potential role of omega-3 fatty acids on MI need to be further explored.

Recently, the Mendelian randomization (MR) method, which regards genetic variation as instrumental variables (IVs) replaced the exposure, has been considered an effective measure to explore the causal effect of the exposure on the outcome (10). Moreover, two-sample MR (TSMR) analysis extracted the summary data of the exposure and the outcome from separate samples, elevating the effectiveness (11).

Exploring the factors affecting MI can not only guide the treatment decision but also provide valuable prognostic information. In this research, we extracted the summary data from published large genetic studies to detect whether there was a causal relationship between omega-3 fatty acids and the risk for MI by TSMR analysis.

## 2 Methods

### 2.1 Data source and instrumental variables selection

We employed the MR design to detect the causal effect of circulating omega-3 fatty acids on the risk of myocardial infarction. Remarkably, the MR study should satisfy the following hypotheses (12): (a) IVs should be strongly related to the exposure, and the *F* statistics will be used to evaluate the association strength; (b) no horizontal pleiotropy exists between IVs and outcome, that the intercept of MR-Egger regression should be non-significant; (c) IVs should not be associated with confounders.

In our study, we regarded single-nucleotide polymorphisms (SNPs) as IVs. SNPs strongly related to circulating omega-3 fatty acids (*P* < 5 × 10^−8^) were extracted from a published genome-wide association study (GWAS) meta-analysis in 2016 containing 13,544 subjects of European ancestry (13). This comprehensive meta-analysis encompassed 14 studies, starting from 1972 and spanning across regions including Finland, Helsinki, the Netherlands, and Germany. To escape the bias caused by strong linkage disequilibrium, we stipulated the inclusion criteria *r*^2^ < 0.001. Meanwhile, we excluded SNPs with palindrome. For the risk of MI, relevant data were abstracted from another GWAS meta-analysis published in 2015 including 171,875 individuals (43,676 cases and 128,199 controls) of European ancestry, incorporating findings from 48 studies (14). Given that EPA and DHA are the primary constituents of omega-3, we investigated the effects of the included SNPs on EPA and DHA levels (15, 16).

### 2.2 Statistical analysis

We employed three MR methods to estimate the casual association between circulating omega-3 fatty acids and myocardial infarction risk: MR-Egger regression, inverse-variance weighted (IVW), and weighted median. The results were presented as odds ratio (OR) with 95% confidence intervals (CIs). The IVW method requests total included IVs to be valid and gives the most accurate estimation (17). However, the weighted median and weighted mode method only requires half of included IVs to be valid (18). The MR-Egger regression method does not force the regression equation to go through the origin and gives a relatively wider CI. Moreover, the intercept of the MR-Egger regression equation was performed to evaluate the existence of the horizontal pleiotropy pathway between IVs and the outcome. If the intercept was not significant, we considered there was no horizontal pleiotropy pathway (19). Subsequently, to assess the stability of our results, we removed SNPs in sequence and employed the IVW method to estimate the effects of the remaining IVs. Then, Cochran's Q statistics was used to estimate the heterogeneity among included SNPs (20). To minimize confounding effects, we used the PhenoScanner website to exclude SNPs that were associated with established risk factors for myocardial infarction (such as hypertension, diabetes mellitus, dyslipidemia, kidney dysfunction, and obesity) from our instrumental variables. We defined two-sided *P *< 0.05 as statistical significance. All statistical analyses were performed by the TwoSampleMR package in the R software (version 3.6.2).

## 3 Results

**Figure 1:**
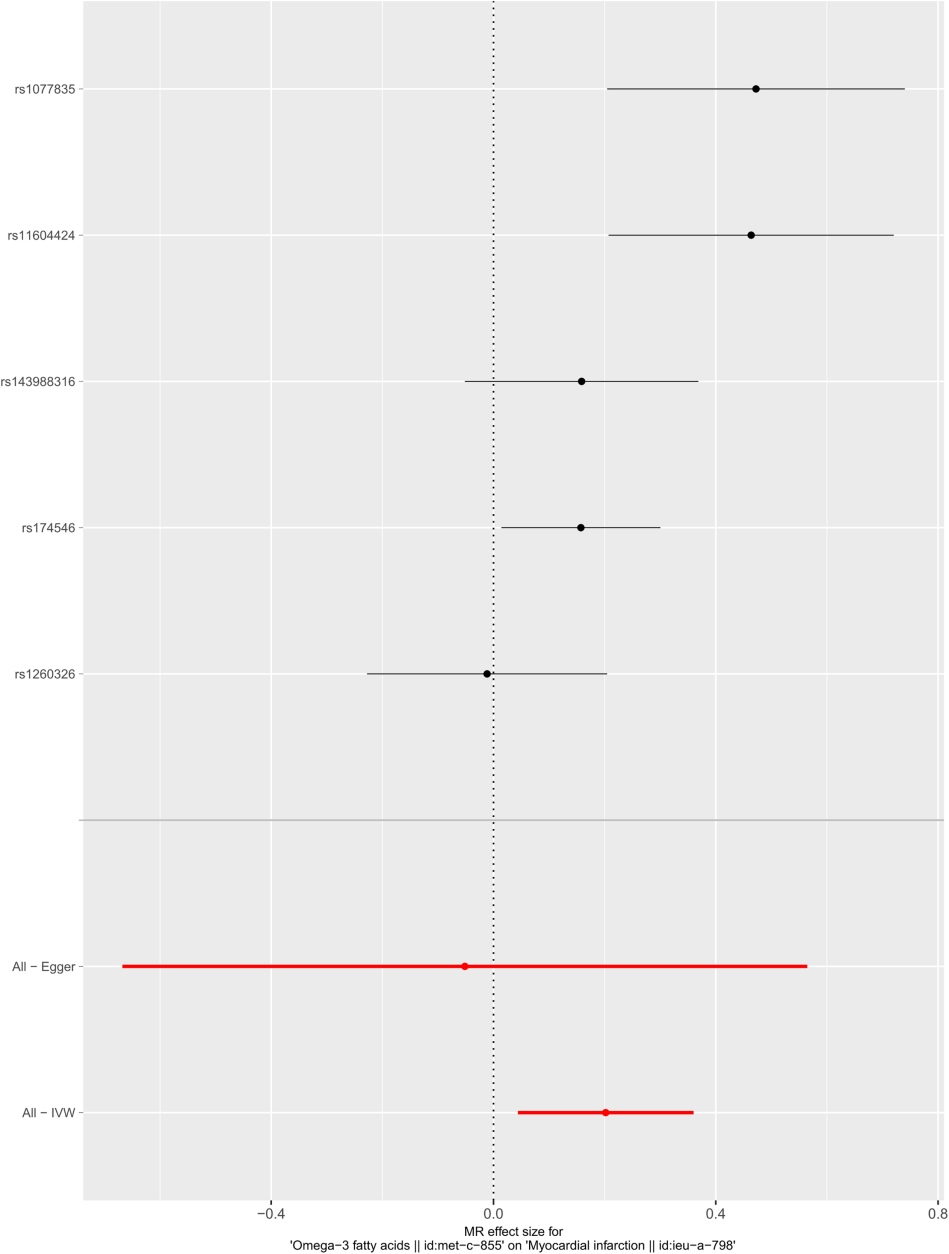
Forest plot of the casual association between circulating omega-3 fatty acids and the risk of myocardial infarction.

**Figure 2:**
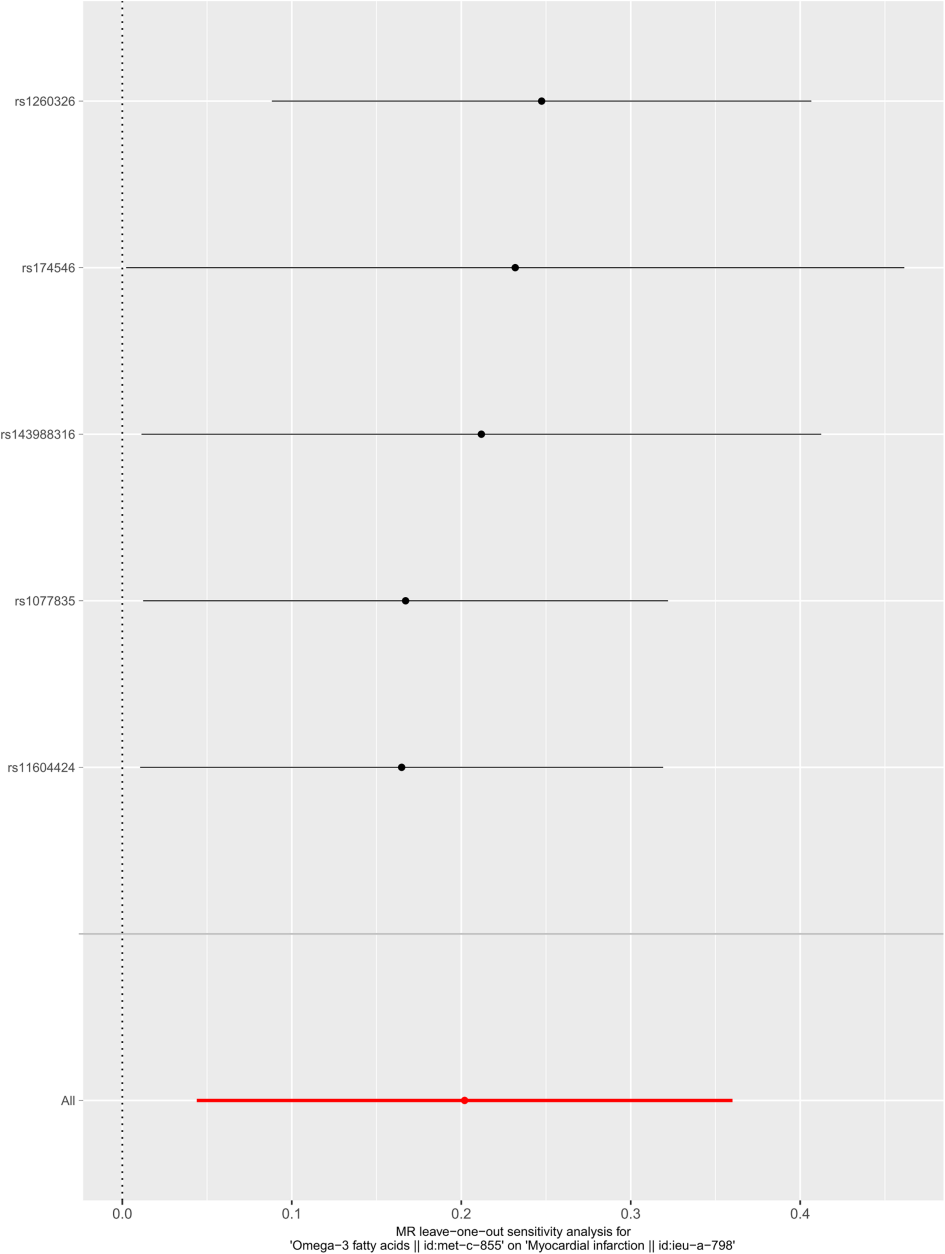
Sensitivity analysis of the casual association between circulating omega-3 fatty acids and the risk of myocardial infarction.

**Figure 3:**
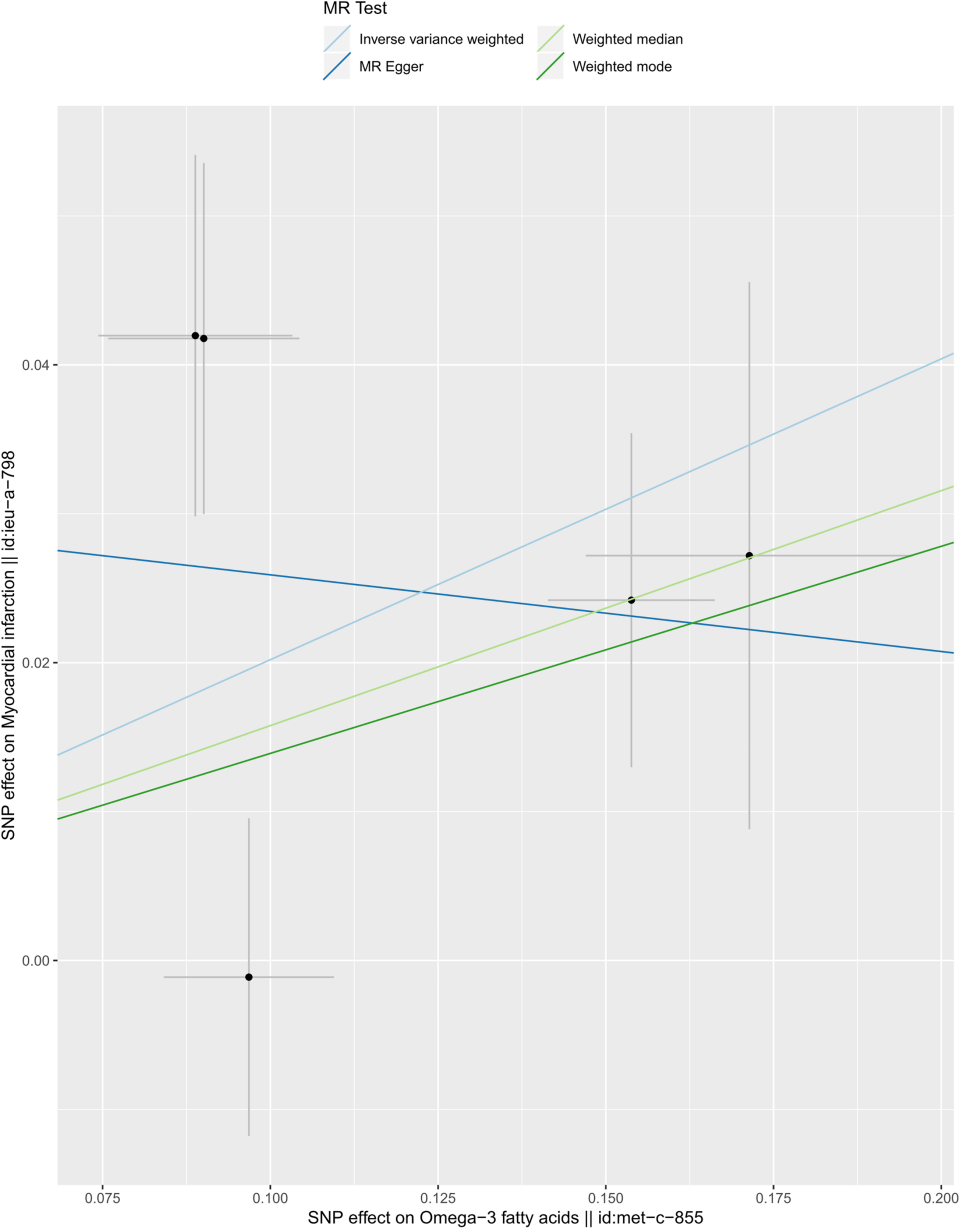
Scatter plot of the casual association between circulating omega-3 fatty acids and the risk of myocardial infarction.

**Figure 4:**
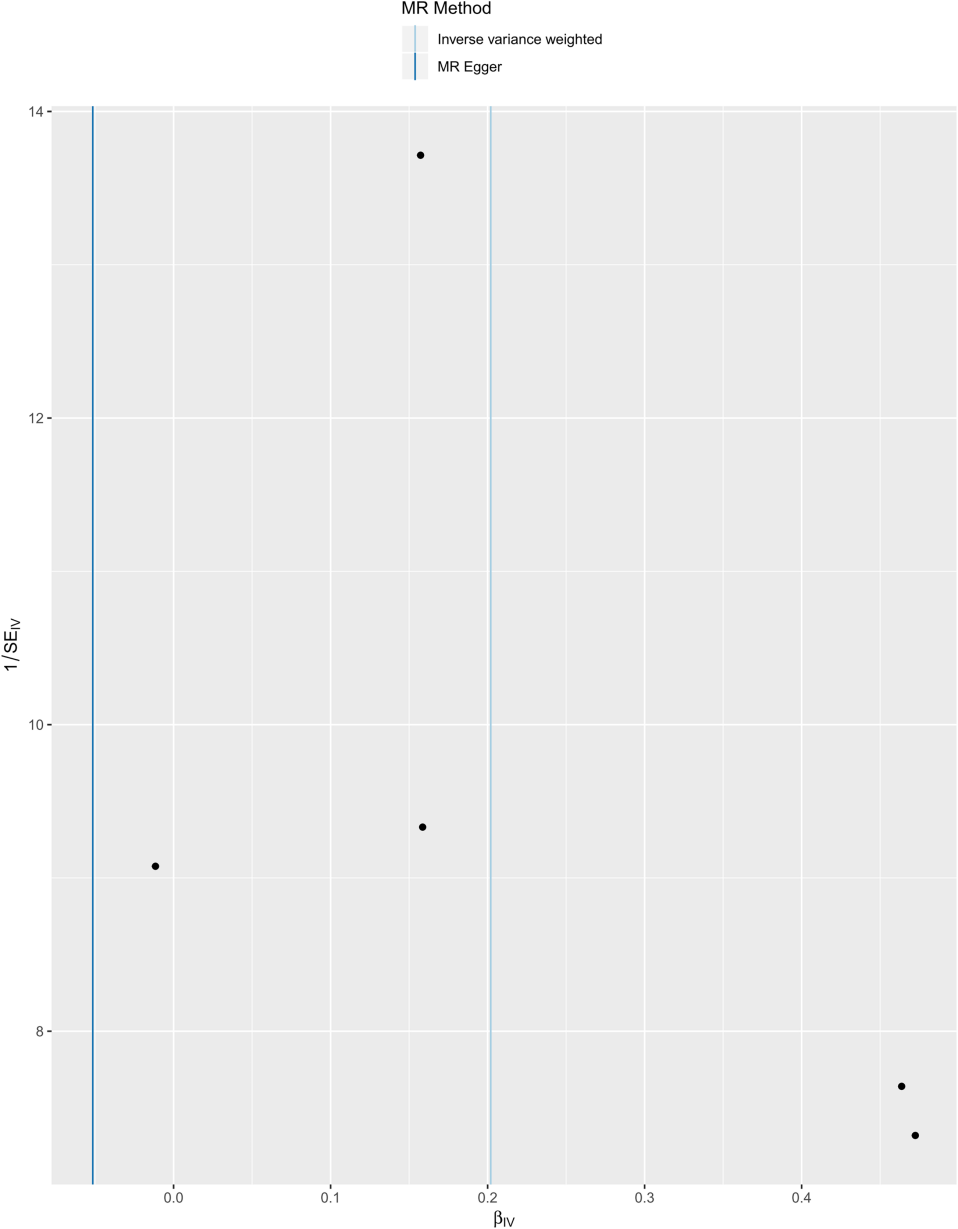
Funnel plot of the casual association between circulating omega-3 fatty acids and the risk of myocardial infarction.

We selected six SNPs that are strongly associated with circulating omega-3 fatty acids (*P* < 5 × 10^−8^). Then, we excluded rs145717049, which was not found in the GWAS outcome. Five SNPs (rs11604424, rs1260326, rs143988316, rs174546, and rs1077835), strongly related to circulating omega-3, were finally included in our study as IVs and are detailed in Table 1 and Supplementary Tables S1, S2. Included SNPs explained 1.27% of the variance in circulating omega-3 fatty acids. We ignored the bias caused by weak IVs, for the *F* statistics far more than 10 (*F* = 34.9). The results of IVW (OR = 1.224, 95% CI = 1.045–1.433, *P* = 0.012), weighted median method (OR = 1.171, 95% CI = 1.042–1.315, *P* = 0.008), and weighted mode (OR = 1.149, 95% CI = 1.002–1.317, *P* = 0.117) showed that genetically predicted lower circulating omega-3 increased the risk of myocardial infarction, although the result of MR-Egger was not significant (OR = 0.950, 95% CI = 0.513–1.760, *P* = 0.880) exhibiting a broader confidence interval (Figure 1). The sensitivity analysis performed by the leave-one-out method confirmed the stability of our results (Figure 2). In addition, the intercept of the MR-Egger regression equation suggested an absence of horizontal pleiotropy between the IVs and the outcome, implying that the IVs are likely to influence the risk of myocardial infarction exclusively through the levels of circulating omega-3 (intercept = 0.031, SE = 0.037, *P* = 0.464) (Figure 3). The funnel plot showed significant heterogeneity among included SNPs (Cochran's Q = 12.211, *P* = 0.016) (Figure 4).

**Table 1:** Genome-wide significant variants of circulating omega-3 fatty acids and their association with myocardial infarction.

SNP	Effect_allele	Other_allele	EAF	Omega-3			Myocardial infarction		
				Beta	SE	*P*	Beta	SE	*P*
rs11604424	T	C	0.756	−0.090	0.014	3.32 × 10^−10^	−0.042	0.012	0.000
rs1260326	C	T	0.637	−0.097	0.013	3.37 × 10^−14^	0.001	0.011	0.917
rs143988316	T	C	0.069	−0.171	0.024	2.95 × 10^−12^	−0.027	0.018	0.139
rs174546	T	C	0.403	−0.154	0.012	1.19 × 10^−34^	−0.024	0.011	0.031
rs1077835	G	A	0.250	0.089	0.014	1.08 × 10^−9^	0.042	0.012	0.001

EAF, effect allele frequency.

## 4 Discussion

The current study performed a Mendelian randomization study to explore the causal relationship between circulating omega-3 and the risk of MI and demonstrated that lower circulating omega-3 increased the risk of MI. However, several studies have showed inconsistent results (21, 22). Del et al. analyzed the data from a global consortium of 19 studies comprised of 16 countries, 45,637 unique individuals, 8,000 first CHD events, and 7,157 non-fatal MI events (21). The results showed that the omega-3 biomarkers DHA, plant-derived α-linolenic acid (ALA), and docosapentaenoic acid (DPA) were related to a lower risk of fatal CHD (21). However, no significant relationships were observed between non-fatal MI and omega-3 biomarkers (21). Another study, which included 68,680 patients and reported 7,044 deaths, 1,150 sudden deaths, 3,993 cardiac deaths, and 1,837 MI, also showed that omega-3 fatty acid supplementation was not related to a lower risk of all-cause mortality and MI (22). Two recent studies also reached a similar result that supplementing omega-3 fatty acids did not reduce the incidence of major cardiovascular events or cancer (23, 24). However, in these studies, the heterogeneity of the subjects and the varying levels of dietary background intake may lead to negative results.

There is, of course, a large body of evidence supporting our findings that omega-3 fatty acids have cardioprotective effects (3, 25–27). The GISSI-Prevenzione trial in 1999 showed that omega-3 decreased the risk of death and non-fatal acute MI in patients with recent MI (<3 months) (28). A meta-analysis of 15,806 CHD patients found that omega-3 fatty acids reduce the risk of non-fatal and fatal MI by 20% and 30%, respectively (29). A study published in 2007 also showed that, in Japanese hypercholesterolaemic patients, EPA may be a promising treatment for preventing major coronary events, especially non-fatal coronary events (30). A meta-analysis performed by Casula et al. included 15,348 patients with a history of CVD and demonstrated that supplementation of a high-dose omega-3 fatty acid had significant protective effects on the onset of MI, sudden death, and cardiac death (31). In 2018, the American Heart Association (AHA) published guidance in support of the dietary intake of fish in primary prevention (32). A multi-center randomized trial, published in 2019, certified that the risk of ischemic events was decreased in the icosapent ethyl group than in the placebo group, even when triglyceride levels were high in the study group (1). A meta-analysis, published in 2019, combined data from 13 RCTs and showed that supplementation of omega-3 reduced the MI risk even after exclusion of REDUCE-IT (33).

Reduced blood pressure, triglyceride levels, and low heart rate variability achieved RR reductions for myocardial infarction (34–36), and platelet oxidative stress leads to increased platelet adhesion to damaged endothelial cells, which contributes to the progression of injury (37). Omega-3 fatty acids have anti-inflammatory properties and may modulate the inflammatory response (38, 39); EPA and DHA can reduce blood pressure (40), platelet aggregation (41), triglyceride (42), heart rate (43), and increase heart rate variability (44). As a consequence, all of these properties can reduce the risk of MI. In the era of precision, medicine may aid in the management of MI.

Although our study first provided a causal association that genetically predicted lower circulating omega-3 fatty acids increases the risk of myocardial infarction, there were some limitations that should be noticed. First, our study only paid attention to subjects of European ancestry, which would limit our results to apply to other race. Second, limited SNPs were selected as IVs in our study, which explained the limited heritable variance of the circulating omega-3 fatty acids. Third, there has not been an appropriate method to precisely evaluate the third assumption of the MR study, which might cause some bias. Finally, although our study theoretically proved the causal relationship between omega-3 fatty acids and myocardial infarction, further population studies are needed to demonstrate this finding.

## 5 Conclusion

This study highlights the potential value of omega-3 fatty acids in reducing the risk of myocardial infarction. These findings provide a significant basis for nutrition-based preventive strategies. Consequently, further clinical trials are essential to validate these preliminary findings and ensure the translation of research into effective dietary guidelines.

## Data Availability

The original contributions presented in the study are included in the article/Supplementary Material, further inquiries can be directed to the corresponding author.
