# Supplementary material for: Decreased circulating omega-3 fatty acids increase the risk of myocardial infarction: a two-sample Mendelian randomization study
Source: Front Cardiovasc Med. 2024 Mar 14;11:1328087. doi: 10.3389/fcvm.2024.1328087 (PMC10972898; doi:10.3389/fcvm.2024.1328087)
Supplement: Supplementary file 1 [file Table1.docx]

| SNPs | Position | Consequence | Gene |
| --- | --- | --- | --- |
|  |  |  |  |
| rs11604424 | chr11:116780399 | Intron Variant | ZPR1 |
| rs1260326 | chr2:27508073 | Missense Variant | GCKR |
| rs143988316 | chr19:19556445 | Non-coding Variant | - |
| rs174546 | chr11:61802358 | 3 Prime UTR Variant | FADS1 |
| rs1077835 | chr15:58431227 | Intron Variant | LIPC |

Table S1 The detailed information of the selected SNPs in this study

Table S2 The correlation between selected SNPs and EPA and DHA.

| SNP | effect_allele | other_allele | EPA | | | DHA | | |
| --- | --- | --- | --- | --- | --- | --- | --- | --- |
|  |  |  | Beta | SE | *P* | Beta | SE | *P* |
| rs11604424 | T | C | - | - | - | -0.028 | 0.005 | 9.8E-09 |
| rs1260326 | C | T | -0.003 | 0.004 | 0.342 | -0.048 | 0.004 | 7.2E-33 |
| rs143988316 | T | C | - | - | - | -0.099 | 0.007 | 5.1E-43 |
| rs174546 | T | C | -0.034 | 0.004 | 3.1E-21 | -0.288 | 0.004 | 7.5E-1074 |
| rs1077835 | G | A | 0.002 | 0.004 | 0.722 | 0.127 | 0.005 | 5.1E-159 |
